# Transcriptomic profiling of subpopulations of mouse embryonic subplate neurons

**DOI:** 10.1111/joa.70197

**Published:** 2026-07-16

**Authors:** Hitomi Achiwa, Yuichiro Hara, Hideya Kawaji, Minori Oshima, Noe Kaneko, Ayumu Morioka, Anna Hoerder‐Suabedissen, Zoltán Molnár, Chiaki Ohtaka‐Maruyama

**Affiliations:** ^1^ Developmental Neuroscience Project Tokyo Metropolitan Institute of Medical Science Tokyo Japan; ^2^ Graduate School of Education The University of Tokyo Tokyo Japan; ^3^ Research Center for Genome & Medical Sciences Tokyo Metropolitan. Institute of Medical Science Tokyo Japan; ^4^ Department of Physiology, Anatomy and Genetics Sherrington Building, University of Oxford Oxford UK

**Keywords:** layer 6b, Lpar1‐EGFP, NeuroD1‐Cre‐ERT2 mouse line, single‐cell transcriptomics, subplate, Visium spatial transcriptomics

## Abstract

Subplate neurons (SpNs) are among the earliest‐born and maturing neurons in the developing cerebral cortex. They arise from multiple origins and can be classified into several subgroups based on morphology, connectivity, and gene expression. These neurons play essential roles in cortical circuit formation, yet their cellular diversity and transcriptional dynamics remain incompletely understood. Here, we characterized transcriptomic profiles of SpN subpopulations in embryonic mouse cortex using *Lpar1‐EGFP* and *NeuroD1/Cre‐ERT2 (D1B)* reporter lines. We applied complementary approaches of gene expression profiling, including bulk microarray analysis, single‐cell RNA sequencing (scRNA‐seq), and Visium spatial transcriptomics. At embryonic day 17 (E17), scRNA‐seq identified 10 distinct *Lpar1‐EGFP*‐positive SpN clusters, which spatial transcriptomics mapped to specific cortical regions. While many markers showed enrichment within the subplate region, others extended into the hippocampus and ventral pallium (including the amygdala, claustrum, and endopiriform nucleus). Integrated analysis of *Lpar1‐EGFP* and D1B lines revealed both overlapping and unique gene expression signatures, highlighting dynamic markers of subplate identity. Comparisons between E15 and E17 datasets showed substantial transcriptional shifts, suggesting rapid developmental changes in SpN subgroups. Validation with in situ hybridization and RNAscope confirmed the selectivity of key markers, including *Cryab*, *Cdh13*, *Nr4a2,* and *Lmo3*. Together, these findings provide a molecular framework for further classifying SpN subtypes and identifying candidate markers for transient versus persistent populations, thereby advancing our understanding of early cortical development.

## Graphical abstract

To study gene expression in subpopulations of cortical subplate neurons (*Lpar1‐EGFP*‐positive and D1B‐positive SpN clusters), we applied gene expression profiling, including bulk microarray analysis, single‐cell RNA sequencing (scRNA‐seq), and Visium spatial transcriptomics, and identified both overlapping and unique gene expression signatures, highlighting dynamic markers of subplate identity. Validation with in situ hybridization and RNAscope confirmed the selectivity of key markers, including *Cryab*, *Cdh13*, *Nr4a2,* and *Lmo3*.

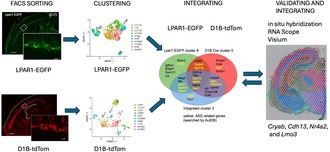

## 1 INTRODUCTION

Subplate (SP) is a transient but critical compartment in mammalian brain development, first described in dog, sheep, and human fetal cortex by Kostović and Molliver in the 1970s (Molliver et al., 1973). It is present in all developing mammalian cerebral cortices and plays essential roles in early circuit formation, neuronal migration, and area specification. Subplate neurons (SpNs) orchestrate the initial wiring of thalamocortical and cortico‐cortical circuits and may also contribute to neurodevelopmental vulnerabilities unique to humans (Allendoerfer & Shatz, 1994; Molnar & Kwan, 2024). In the mouse, some embryonic SpNs are transient, but a considerable subset persists as layer 6b neurons in adulthood. These neurons exhibit unique sensitivities to neuromodulators and form distinctive intracortical and thalamic projections (Bayer et al., 2004; Case & Broberger, 2018; Feldmeyer, 2023; Messore, Narayanan Therpurakal, et al., 2025; Messore, Vadisiute, et al., 2025; Zolnik et al., 2024). Recent transcriptomic studies have provided refined classifications of adult cortical neuronal subtypes, including layer 6b glutamatergic clusters (Feldmeyer, 2023; Yang et al., 2022; Yao et al., 2021). Such molecular signatures are validated against classical anatomical criteria such as dendritic morphology, projection patterns, and electrophysiological properties. Approaches ranging from layer‐specific dissections (Bakken et al., 2016; Belgard et al., 2011), single‐cell transcriptomics (Sugino et al., 2006), and back labeling combined with sorting and single‐cell transcriptome analysis, also called retro‐seq (Tasic et al., 2018), have been used. These studies are gradually converging on a consensus taxonomy of adult cortical cell types (Gao et al., 2025; Henning et al., 2023; Nano et al., 2025; Yao et al., 2023).

In contrast, developmental gene expression patterns are far more dynamic. The SP zone contains both resident neurons and cell types migrating through the SP zone, complicating molecular classification. SpNs undergo extensive changes in gene expression, morphology, connectivity, and density during cortical expansion, with many undergoing selective cell death. The extent of such cell loss in rodent SP remains debated (Price et al., 1997; Valverde et al., 1995). Birthdating and marker studies indicate that while SpN density within the cerebral cortex declines sharply after birth, a subset survives as layer 6b neurons, defined by specific molecular markers (Hoerder‐Suabedissen & Molnar, 2013).

SpNs are heterogeneous in origin. Some arise directly or indirectly from cortical radial and intermediate progenitors (Vasistha et al., 2015), while others, including subsets of glutamatergic and GABAergic neurons, migrate tangentially from extracortical sources (Boon et al., 2019; Pedraza et al., 2014). Developmental expression of lineage‐defining genes, such as *Lhx6* in GABAergic precursors or *Satb2* in upper‐layer projection neurons, underscores the predictive value of transcriptional signatures even before terminal differentiation. However, embryonic gene expression often diverges from postnatal and adult patterns (Hara et al., 2025); only a few markers, such as Cplx3, Ctgf, and Nurr1/Nr4a2, persist across stages (Belgard et al., 2011; Hoerder‐Suabedissen et al., 2009; Hoerder‐Suabedissen & Molnar, 2013; Oeschger et al., 2012). This dynamism complicates attempts to align embryonic SP identity with adult classifications (Henning et al., 2023).

To date, studies of SP and layer 6b transcriptomes have largely analyzed heterogeneous tissue samples, without resolving distinct neuronal subtypes (Hoerder‐Suabedissen et al., 2009; Hoerder‐Suabedissen & Molnar, 2013; Oeschger et al., 2012). Reporter mouse lines, such as *Lpar1‐EGFP, Ctgf‐EGFP, Golli‐tau‐EGFP*, and *Drd1a‐Cre*, enable more precise targeting of subpopulations. However, most of these existing lines have been confirmed to show expression only in postnatal L6b neurons. In contrast, *Lpar1‐EGFP* labels embryonic SpNs and has been extensively characterized, marking subsets of excitatory SpNs as well as GABAergic neurons, including Lhx6+/Som + interneurons (Ghezzi et al., 2021; Hoerder‐Suabedissen & Molnar, 2013; Marques‐Smith et al., 2016).

Morphologically, Lpar1‐EGFP SpNs comprise fusiform neurons with local axons and pyramidal neurons projecting through the marginal zone (Ghezzi et al., 2021). Functional studies demonstrated that the fusiform SpNs receive translaminar input until the emergence of whisker barrels, coincident with cell death, whereas pyramidal SpNs gradually acquire input from overlying cortical layers. Sparse direct thalamic innervation has also been demonstrated (Ghezzi et al., 2021).

To investigate the gene expression profile of SpN during embryonic development in detail, a system for labeling SpN from an early stage is essential. However, no such system existed except for the *Lpar1‐EGFP* strain. Hirata et al. developed neurogenic‐tagging mouse strains that label cell populations differentiating concurrently during development using various promoters (Hirata et al., 2021). Among these, the NeuroD1‐CreERT2 (D1B) driver mouse line, crossed to a tdTomato reporter strain (Ai14), enabled specific and early labeling of SpN through tamoxifen administration between E10.5 and E11.5. In this study, we applied three complementary transcriptomic approaches to characterize embryonic SP populations in *Lpar1‐EGFP* and *NeuroD1/CreERT2* (D1B) reporter lines. First, we profiled FACS‐sorted Lpar1‐EGFP neurons at E15 and E18 using microarray and compared them with previously reported SP markers (Belgard et al., 2011; Hoerder‐Suabedissen et al., 2009; Hoerder‐Suabedissen & Molnar, 2013; Oeschger et al., 2012). Second, we performed single‐cell RNA sequencing (scRNA‐seq) of both reporter lines (*Lpar1‐EGFP* and *NeuroD1/Cre‐ERT2 (D1B)*) at E17 to resolve neuronal heterogeneity. Third, we integrated these datasets with Visium spatial transcriptomics to map molecular profiles onto cortical architecture. By combining these methods with publicly available datasets, we define transcriptional signatures of embryonic SpNs, identify dynamic gene expression changes, and provide a molecular framework for exploring the diversity of SP subtypes during cortical development.

## 2 MATERIALS AND METHODS

### 2.1 Animals

#### 2.1.1 *Lpar1‐EGFP* mouse line

*Tg(Lpar1‐EGFP)GX193Gsat* was obtained from MMRRC. The *Lpar1‐EGFP* line has been previously characterized by Hoerder‐Suabedissen & Molnar (2013), Marques‐Smith et al. (2016), Ghezzi et al. (2021).

All animals were treated in accordance with the Tokyo Metropolitan Institute of Medical Science Animals Care and Use Committee guidelines. Wild‐type (WT) ICR females (supplied by Japan SLC) were mated for 12 h overnight with *Lpar1‐EGFP* males [*Tg(Lpar1‐EGFP)GX193Gsat*, on an NIHS background] and checked for plugs. Midday after the mating was considered E0.5. Timed pregnant ICR females were killed by cervical dislocation at midday on E15 (*N* = 55 embryos from 9 pregnant mothers) and E18 (*N* = 47 embryos from 8 pregnant mothers), the embryos were removed, brains were dissected out, and washed in ice‐cold 0.1 M phosphate‐buffered saline (PBS) for microarray analysis.

GFP expression is driven from a genomically integrated copy of bacterial artificial chromosome (BAC) RP23‐149O20, in which GFP is inserted into the first codon of the *lpar1/edg2* gene. Based on both GFP expression and GFP‐genotyping results, female littermates are always GFP‐negative (i.e., WT; *n* > 250), suggesting that the GFP‐containing BAC is inserted into the y‐chromosome. GFP is evident in E13.5 brains and labels cells in the ventricular zone (VZ) and preplate. By E15.5, the EGFP+ cells are located both in the marginal zone and in the SP, but are absent from the cortical plate.

#### 2.1.2 *NeuroD1‐CreERT2 (D1B)* mouse line

SpN‐Specific Cre‐ERT2 mouse line—*NeuroD1‐CreERT2(D1B)* driver mouse line using the *neuroD1* enhancer was obtained from the Hirata laboratory at the National Institute of Genetics. Hirata et al. produced neurogenic tagging mouse lines, which can be used to assign *CreER*‐*loxP* recombination to neuron subsets that share the same differentiation timing in living animals and enable various experimental manipulations of the classified subsets. We performed scRNA‐seq analysis using transgenic mouse lines that label distinct SpN cell populations. The *NeuroD1‐Cre ERT2(D1B)* driver line labels SpNs if administered with tamoxifen at E10.5 (Hirata et al., 2021).

To visualize Cre‐mediated recombination, the *NeuroD1‐CreERT*2 mice were crossed with a Cre‐dependent reporter line. The *Ai14* reporter mouse line (*Rosa26‐CAG‐LSL‐tdTomato*; Madisen et al., 2010) was used as a Cre‐dependent fluorescent reporter. In this line, a loxP‐flanked STOP cassette prevents tdTomato expression until Cre‐mediated recombination occurs, enabling robust labeling of Cre‐expressing cells and their progeny. Timed pregnant *D1B/Ai14* females were killed by cervical dislocation at midday on E17 (*N* = 8 embryos from 2 pregnant mothers); the embryos were removed, brains were dissected, and washed in ice‐cold 0.1 M PBS for FACS sorting.

### 2.2 Cell sorting

The method was performed as described previously (Ohtaka‐Maruyama et al., 2018). For Lpar1‐EGFP brains, the meninges were removed prior to further processing. Briefly, after isolating the dorsal cortex from both hemispheres of *Lpar1‐EGFP* mouse or *D1B/Ai14* mouse embryonic brains at E15, E17, and E18 for Lpar1‐EGFP and E17 for D1B/Ai14, EGFP or tdTomato positive cells were collected as follows. The tissue fragments were treated with 8–10 U/mL papain (Worthington Biochemical) and 10 μg/mL DNase I (TAKARA Bio) and then triturated by pipetting in 10% foetal Calf Serum (FCS)/ Dulbecco's modified Eagle's medium (DMEM). Dissociated cortical cells were collected by centrifugation (1000× ***g*** for 5 min) and then re‐suspended in HEPES‐buffered saline (HBS) at a concentration of 1–2×10^6^ cells/mL. The cell solution was fed through fluorescence‐activated cell sorting (FACS). Cells expressing EGFP or tdTomato were sorted using a BD FACSAria III (BD Biosciences) or Sony SH800 cell sorter (Sony). Cell debris and aggregates were excluded based on forward and side scatter profiles, followed by exclusion of dead cells using propidium iodide (for EGFP‐positive samples) or 7‐AAD (for tdTomato‐positive samples). EGFP‐positive cells were identified based on green fluorescence (488 nm excitation), whereas tdTomato‐positive cells were detected using appropriate red fluorescence channels (561 nm excitation, 581 nm emission filter, FL3). Cells prepared from brains lacking EGFP expression were used as negative controls to define gating thresholds, and only live, fluorescently labeled cells were collected for downstream analysis.

### 2.3 Microarray analysis of gene expression of FACS‐sorted Lpar1‐EGFP subplate neurons at E15 and E18

Total RNA was extracted from the collected EGFP‐positive cells (~2 × 10^5^ cells) using Trizol (Invitrogen) according to the manufacturer's protocol, and microarray analysis was performed with the whole cortex as a control. The quality of RNA was assessed with a 2100 Bioanalyzer (Agilent Technologies). Cy3‐labeled cRNA was prepared using a Low Input Quick Amp Labeling Kit according to the manufacturer's protocol (Agilent Technologies). Samples were hybridized to the Mouse Gene Expression v2 Microarray (G4846A, Agilent Technologies), washed, and then scanned using a SureScan Microarray Scanner (Agilent Technologies). The microarray images were analyzed with Feature Extraction software (Agilent Technologies). Three independent microarray experiments were performed using independently prepared EGFP‐positive cells and cells isolated from the whole cortex.

Before analyzing gene expression, microarray data were normalized and processed using GeneSpring GX 14.5 software (Agilent Technologies). Signal intensities were normalized using a 75th percentile shift normalization. Control and low‐quality probes were removed, and probes with low expression levels were filtered out as part of quality control.

Differentially expressed genes were identified using *t*‐tests implemented in GeneSpring, and *p* values were corrected for multiple testing using the Benjamini–Hochberg false discovery rate (FDR) method. Genes with an FDR‐adjusted *p* value < 0.05 were considered statistically significant.

### 2.4 iHop

iHop (biotools:ihop) is an online resource (https://bio.tools/ihop) that allows users to explore a network of gene and protein interactions based on published scientific literature. For each gene search, it reports sentences from abstracts associating it with other genes, links out to full abstracts, and reports experimental evidence for the interactions, if available. We used this to identify interacting partners of proteins encoded by genes with SP‐enriched expression.

### 2.5 Comparisons with previously published datasets

Transcriptomic datasets, including microarray‐based data (Hoerder‐Suabedissen et al., 2009; Hoerder‐Suabedissen & Molnar, 2013; Oeschger et al., 2012) and sequencing‐based data (Belgard et al., 2011), derived from dissected SP/layer 6b, were analyzed to compare the current gene expression profiles with those reported in the Subplate Gene Expression Atlas (https://subplate.schoolofdata.ch) (Belgard et al., 2011; Hoerder‐Suabedissen & Molnar, 2013).

### 2.6 Allen Brain Institute resources

Each gene identified by the Lpar1‐EGFP+ FACS sorted microarray but absent from the published list of SP‐enriched gene expression (Hoerder‐Suabedissen & Molnar, 2013) was checked against the available images in the Allen Developing brain gene expression atlas. Genes with visually confirmed expression in the SP compartment were followed up for further investigation and validation.

### 2.7 C1 single‐cell RNA sequencing

EGFP‐positive cells (4 × 10^6^) were isolated by FACS sorting from 10 embryonic E17 cortices derived from 3 mothers of *Lpar1‐EGFP* mated mice for each experiment. We conducted the same experiment twice.

For td‐Tomato positive cells, 7259 td‐Tomato positive cells were isolated by FACS sorting from 8 embryonic E17 cortices derived from 2 mothers of *D1B/Ai14* mice. The sorted cell suspension was adjusted to 400 cells/μL, and 15 μL (total 6000 cells) was loaded into an Integrated Fluidic Circuit (IFC) (10–17 μm; Fluidigm, Inc.). After cell loading, single cells captured in individual chambers of IFCs were manually confirmed using an inverted microscope (Keyence). Cell lysis, total RNA isolation, cDNA synthesis, and pre‐amplification of synthesized cDNAs were performed using the C1 script of “mRNA Seq HT:RT & (1912x)” according to the manufacturer's protocol (PN 10‐14964; Fluidigm, Inc.). During cDNA pre‐amplification, individual cells were pre‐barcoded at the 3′‐end cDNAs with 40 different Fluidigm cell‐specific indices. Following the completion of pre‐amplified cDNA preparation in the C1 systems, individual pre‐indexed cDNA samples, including External RNA Controls Consortium (ERCC) spike‐in (Invitrogen) that was pre‐diluted, were transferred from an IFC to a regular 96‐well polymerase chain reaction (PCR) plate. For dual indexing, 20 different i7 barcode‐containing primers in Nextera XT index Kit v2 Set A/B (Illumina, Inc.) were annealed to the 5′‐end of fragmented cDNAs following tagmentation of individual cDNA samples. We constructed 1600 dual‐indexed and 3′‐end enriched cDNA libraries (400 cDNA libraries per cell line) using Nextera XT DNA library preparation kits (Illumina, Inc.). Ten cDNA library pools (40 libraries per cDNA library pool) per cell line were individually quantified using Qubit assay (Invitrogen), and further quality and quantity checks of the cDNA library pools were performed in 2100 Bioanalyzer systems (Agilent, Inc.). Based on the molarity measured, individual cDNA library pools were equimolarly combined and sequenced in the RIMD NGS core facility, Osaka University (Osaka, Japan) using HiSeq 3000 and NovaSeq 6000 systems (Illumina, Inc.) with 150 bp paired‐end sequencing.

### 2.8 Data processing and clustering

Sequencing reads were aligned to the *Mus musculus* reference genome (mm10) using STAR (v2.7.6a), guided by GENCODE annotation(vM25). Subsequent gene expression quantification was performed with feature Counts (2.0.6), which provided an exon‐level counting and gene‐level summarization. The gene expression profiles of the Lpar1 and D1B samples were processed using the Seurat v3.2.0 platform. To ensure high‐quality data for downstream analysis, we initially filtered the raw count matrices to include only genes detected in at least three cells and cells with a minimum of 200 features. We further refined the dataset by excluding cells with a mitochondrial gene content of 10% or higher. The read count matrix was normalized, and the data sets were integrated using the anchor‐based approach with the Seurat FindIntegrationAnchors and IntegrateData functions. The spots of the three samples were grouped based on their expression profiles by employing the Shared Nearest Neighbor (SNN) approach using the Seurat FindNeighbors and FindClusters functions with an SNN modularity optimization algorithm. We selected a resolution parameter of 0.6 for the Lpar1 dataset, 0.8 for the D1B dataset, and 0.3 for the integrated dataset, respectively, to obtain optimal clustering results. Uniform Manifold Approximation and Projection (UMAP) was performed for dimension reduction of the single‐cell gene expression profiles using the Seurat RunUMAP function. The 2D UMAP plots were visualized using the Seurat DimPlot function and 10× Genomics Loupe Browser v.9.0.0. Marker genes, those with significantly higher expression levels in a cluster than the others, were detected using the Seurat FindMarkers. Clusters were manually annotated, referring to “known” marker genes (Table 1) that were included in those that were automatically inferred.

**TABLE 1:** Cluster marker genes and annotations for each single‐cell RNA‐seq dataset (A: Lpar1‐eGFP, B: D1B‐CreERT2, C: Integrated data).

Cluster	Marker	Cell type
**A**		
LP_cl.0	Zbtb20, Nrp1	CTX1 (cortex)
LP_cl.1	Eomes/Tbr2, Ngn2	IP (intermediate progenitor)
LP_cl.2	Pantr1, Ezr	CTX2
LP_cl.3	Mef2c, Dab1	CTX3
LP_cl.4	Top2a, Mki67	VZp (ventricular zone progenitor)
LP_cl.5	Crym, Nr4a3	HP (hippocampus)
LP_cl.6	Lhx6, Gad1	GABA (GABA neuron)
LP_cl.7	Ccn1, Igfbp5	ChPx (choroid plexus)
LP_cl.8	Igfbp3, Nr4a2	SP (subplate)
LP_cl.9	Lipe, Mrps25	Miscellaneous (Misc)
**B**		
BT_cl.0	Mef2c, Lmo3	SP
BT_cl.1	Nrp1, Zbtb18	CTX1
BT_cl.2	Reln, Calb2	CR (Cajal–Retzius cells)
BT_cl.3	Nes, Eml1	NPC (neural progenitor cell)
BT_cl.4	Vegfa, Ddah1	EC (endothelial cell)
BT_cl.5	Nts, Rgs8	GABA
BT_cl.6	Cd300a, Bace1	MG (microglia)
BT_cl.7	Sema4g, Robo1	YN (young neuron)
BT_cl.8	Nr4a2, Bhlhe22	CTX2
BT_cl.9	Ttl, Mmp17	CTX3
**C**		
Int_cl.0	Eomes/Tbr2, NeuroD1	IP
Int_cl.1	Map1b, Ncam1	CTX
Int_cl.2	Top2a, Mki67	VZp
Int_cl.3	Hs3st4, Nr4a2	SP
Int_cl.4	Reln, Cxcl12	CR
Int_cl.5	Lhx6, Gad1	GABA

*Note*: Cluster marker genes and annotation for each single‐cell analysis data. A: Lpar1‐eGFP dada. B: D1B data. C: integrated data.

### 2.9 Integration with Visium spatial gene expression data

The Visium spatial expression profiles of developing mouse brains were obtained from Hara et al. (2025). The feature‐barcode matrixes were loaded using the Read10X function in Seurat. The expression values of the spots of the E17 slice were normalized by employing the SCTransform approach using the Seurat SCTransform function. These spots were then clustered based on gene expression profiles using the Seurat FindNeighbors and FindClusters functions, and dimension reduction of the gene expression profiles was performed using the Seurat RunPCA and RunUMAP functions. The integration of the scRNA‐seq dataset into the spatial transcriptome one was performed using the Seurat FindTransferAnchors function, where an anchor‐based approach was performed by querying spatial gene expression profiles and referring to single‐cell gene expression profiles. The integrated data set was further processed using the Seurat TransferData function, which yielded a prediction score (a confidential index of cell‐type prediction) for the individual single‐cell clusters at every spot. We chose the single‐cell clusters that exhibited high prediction scores in the spots corresponding to the SP region. The prediction scores were visualized by overlaying them onto the Visium spot using the Seurat SpatialFeaturePlot function (Figures 3 and 6).

### 2.10 Alluvial analysis of cluster correspondence across spatial and single‐cell datasets

Alluvial plots were generated using the R package ggalluvial (v.0.12.5) to evaluate cluster correspondence across spatial and single‐cell transcriptomic datasets. For Visium integration, prediction scores were computed for each spot using reference scRNA‐seq datasets (integrated: resolution = 0.3; Lpar1: resolution = 0.6), and the highest‐scoring cluster was assigned. Predicted clusters were placed on the left axis and Visium clusters (24 categories) on the right, with axis heights and flow widths proportional to spot numbers (Figures 3a, 6a). Clusters 5 and 12 were excluded as they were absent from the analyzed section. For scRNA‐seq comparisons, pre‐integration clusters (Lpar1: resolution = 0.6; D1B: resolution = 0.8) were placed on the left axis and integrated clusters (resolution = 0.3) on the right. Axis heights and flow widths were proportional to cell numbers (Figure 7), whereas Visium‐based alluvial plots were proportional to total spot numbers (Figures 3a, 6a).

### 2.11 In situ hybridization (ISH) and RNA scope

Coronal frozen sections from wild‐type ICR mouse embryonic brains (E17) were used for these experiments. ISH was performed using digoxigenin‐labeled riboprobes as previously described (Ohtaka‐Maruyama et al., 2007). The *Cryab* RNA probe was generated by cloning a PCR‐amplified cDNA fragment into the pBluescript vector. Briefly, first‐strand cDNA was synthesized from total RNA isolated from E17 mouse cortex and used as a template for PCR amplification of the *Cryab* fragment. The PCR product was cloned into the pBluescript vector, and digoxigenin‐labeled antisense RNA probes were synthesized using T7 RNA polymerase.

Detection of *Nr4a2, Cdh13*, and *Lmo3* mRNA **i**n Figure 7b and *Crya*b and *Ctgf* mRNA in Figure 8b was performed using the RNAscope™ Multiplex Fluorescence V2 kit (Advanced Cell Diagnostics) according to the manufacturer's protocol, together with fluorescein, Cy3, and Cy5 TSA fluorophores (Akoya Biosciences).

Briefly, cortical sections (20 μm) were prepared using a cryostat and stored at −80°C until use. Coronal brain sections were first incubated at 60°C for 30 min, fixed in 4% paraformaldehyde in PBS on ice, washed in 100% ethanol, and treated with hydrogen peroxide for 10 min at room temperature. After washing with water, sections were boiled for 5 min in the target retrieval buffer (Advanced Cell Diagnostics). Following target retrieval, sections were washed in water, rinsed in 100% ethanol, and air‐dried overnight. Sections were then treated with protease at 40°C for 10 min, followed by hybridization with probes (Advanced Cell Diagnostics) at 40°C for 2 h prior to signal amplification. Fluorescent signals were further amplified using the TSA Plus system (Akoya Biosciences) before detection. Finally, sections were washed, counterstained with 4',6‐diamidino‐2‐phenylindole (DAPI) for 2 min, and mounted using Fluoro‐KEEPER antifade reagent (Nacalai Tesque).

Images were acquired using a Stellaris 5 confocal microscope (Leica Microsystems).

### 2.12 AutDB

To ensure a comprehensive screening, the full list of human genes was retrieved from AutDB (https://www.mindspec.org/autdb.html) by selecting “View All” under the “Search fields for Human Gene” section. This list was then cross‐referenced with our gene set, and genes associated with ASD in the “Associated Disorder” field were highlighted in yellow.

## 3 RESULTS

Our previous work provided some characterization of the Lpar1‐EGFP mouse line at P8. Some of the previously identified SP markers (Ctgf, Cplx3, Nurr1) were partially co‐localized with this cell population (Hoerder‐Suabedissen et al., 2009; Hoerder‐Suabedissen & Molnar, 2013). To identify earlier gene expression markers for these L6b populations, we sorted the EGFP‐positive cells using FACS at E15 and E18 and used microarray analysis.

### 3.1 Microarray analysis of Lpar1‐EGFP‐positive SP cells sorted by FACS at E15 and E18

**FIGURE 1:**
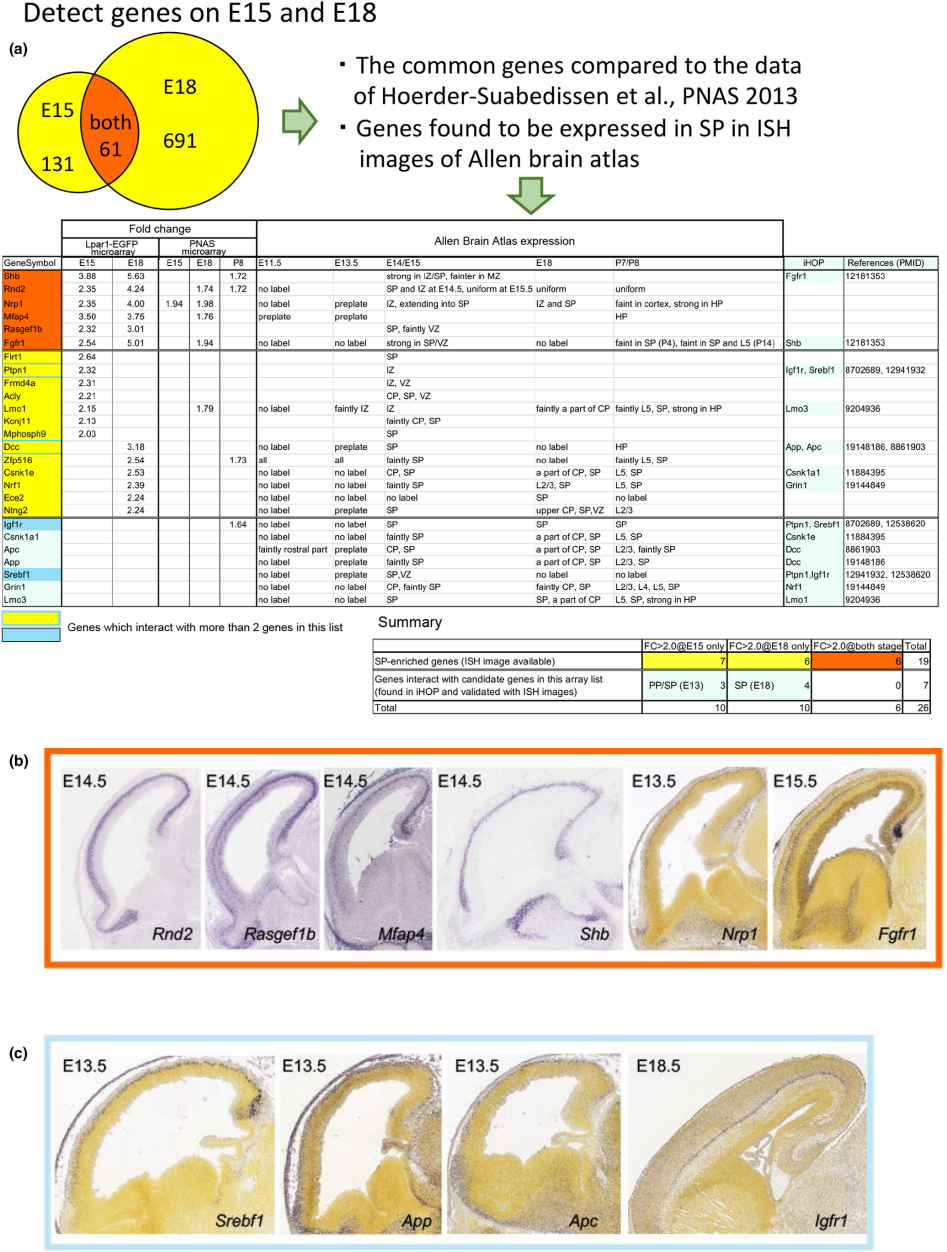
Microarray analysis of Lpar1‐EGFP positive subplate cells sorted by fluorescence‐activated cell sorting (FACS). After isolating the dorsal cortex of Lpar1‐EGFP mouse embryonic brains at two stages (E15 and E18), cells were dissociated. GFP‐positive cells were collected by FACS sorting, RNA was isolated, and microarray analysis was performed with the whole cortex as a control. (a) Venn diagram of the number of genes expressed in GFP‐positive cells that have a fold change of more than two times relative to the entire cortex at E15 or E18. From this list, those in common with previously reported SP‐expressed genes(Hoerder‐Suabedissen et al., 2013) were extracted, and those whose expression could be confirmed by the ISH database were tabulated (26 genes were extracted). Yellow: Genes with FC > 2.0 in only one stage. Orange: Genes with FC > 2.0 at both stages. Blue: Genes extracted by iHOP analysis (validated with ISH images). Thick blue: Genes interacting at the protein level with two or more genes in this list. The table provides a summary of the number of genes in each category. Genes identified by iHOP are shown with their expression patterns in the preplate/subplate (PP/SP) at E13 and in the subplate (SP) at E18. (b) ISH images of genes with FC > 2.0 in both stages, obtained from publicly available datasets (Allen Brain Atlas and GenePaint database). (c) Genes were identified through iHOP analysis. Images were obtained from Allen Brain Atlas.

We selected the EGFP‐expressing subgroups of SpNs from the dorsal cortex of Lpar1‐EGFP mouse embryonic brains at E15 and E18 using FACS sorting. RNA was isolated, and microarray analysis was performed using RNA extracted from the entire unsorted cortex as a control. We identified genes expressed in EGFP‐positive cells showing a > 2‐fold increase compared with the whole cortex at either E15 or E18. At E15 and E18, 131 and 691 genes were identified, respectively, with 61 expressed at both stages (Figure 1a). We then compared these genes with previously reported SP‐expressed genes from microdissected SP/layer 6b tissue (Hoerder‐Suabedissen et al., 2013). Among the overlapping genes, 26 showed consistent expression patterns in the Allen Mouse Brain Atlas ISH database and are summarized in Figure 1a. In the table, yellow indicates genes with FC > 2.0 in only one stage including *Csnk1e, DDC, Ece2, Nrf1, Ntng2, Zfp516, Flrt1, Ptpn1, Lmo1, Frmd4a, Acly, Kcnj11*, and *Mphosph9* (7 at E15 and 6 at E18). Orange indicates genes (*n* = 6) with FC > 2.0 at both stages including: *Rnd2, Rasgef1b, Mfap4, Shb, Nrp1* and *Fgfr1*. Blue indicates genes extracted by iHOP analysis (validated with ISH images). iHOP analysis identified seven genes (*Igf1r, Csnk1a1, Apc, App, Srebf, Grin1*, and *Lmo3*) that interact with one or more genes in the list. Thick blue indicates genes (*n* = 4) interacting at the protein level with two or more genes in this list, including *Ptpn1, Dcc, Igf1r*, and *Srebf1*. To validate the SP‐enriched genes, we examined the publicly available ISH data from the Allen Mouse Brain Atlas for genes showing > 2.0‐fold change at both E15 and E18. *Rnd2, Rasgef1b, Mfap4, Shb, Nrp1*, and *Fgfr1* showed enrichment within the SP region at embryonic stages in the Allen Brain Atlas datasets (Figure 1b). These were all expressed within the SP compartment, although expression was not strictly confined to this region. Several of these genes were also expressed in adjacent structures, including the hippocampus, and *Rnd2* and *Rasgef1b* additionally showed expression in the olfactory bulb. Figure 1c shows the expression patterns of the four genes (*Srebf1*, *App*, *Apc*, and *Igf1r*) identified by the iHOP analysis as being expressed in the preplate and SP. We examined the regions of expression of 26 selected genes within the neocortex at various developmental stages in the ISH database and explored the most common combinations in the expression patterns (Table in Figure 1a). We identified very few genes that have continuous expression in the SP from embryonic to postnatal stages. A few genes that were expressed in SP at E11.5 were also expressed in VZ, intermediate zone (IZ) at early stages, and they were also expressed in the cortical plate at E18.5. Many genes have strong expression in the hippocampus at postnatal stages. At later stages (P4, P56), some of the SP‐enriched genes were also expressed in layer 5. Similar patterns of co‐expression had been previously reported in studies identifying SP‐enriched gene expression from microdissected tissue samples (Hoerder‐Suabedissen & Molnar, 2013).

To investigate the gene expression status at the single‐cell level in more detail, we next performed single‐cell RNA‐seq analysis of SpNs.

### 3.2 Single‐cell RNA seq analysis of Lpar1‐EGFP‐positive cells in E17 dorsal cortex

**FIGURE 2:**
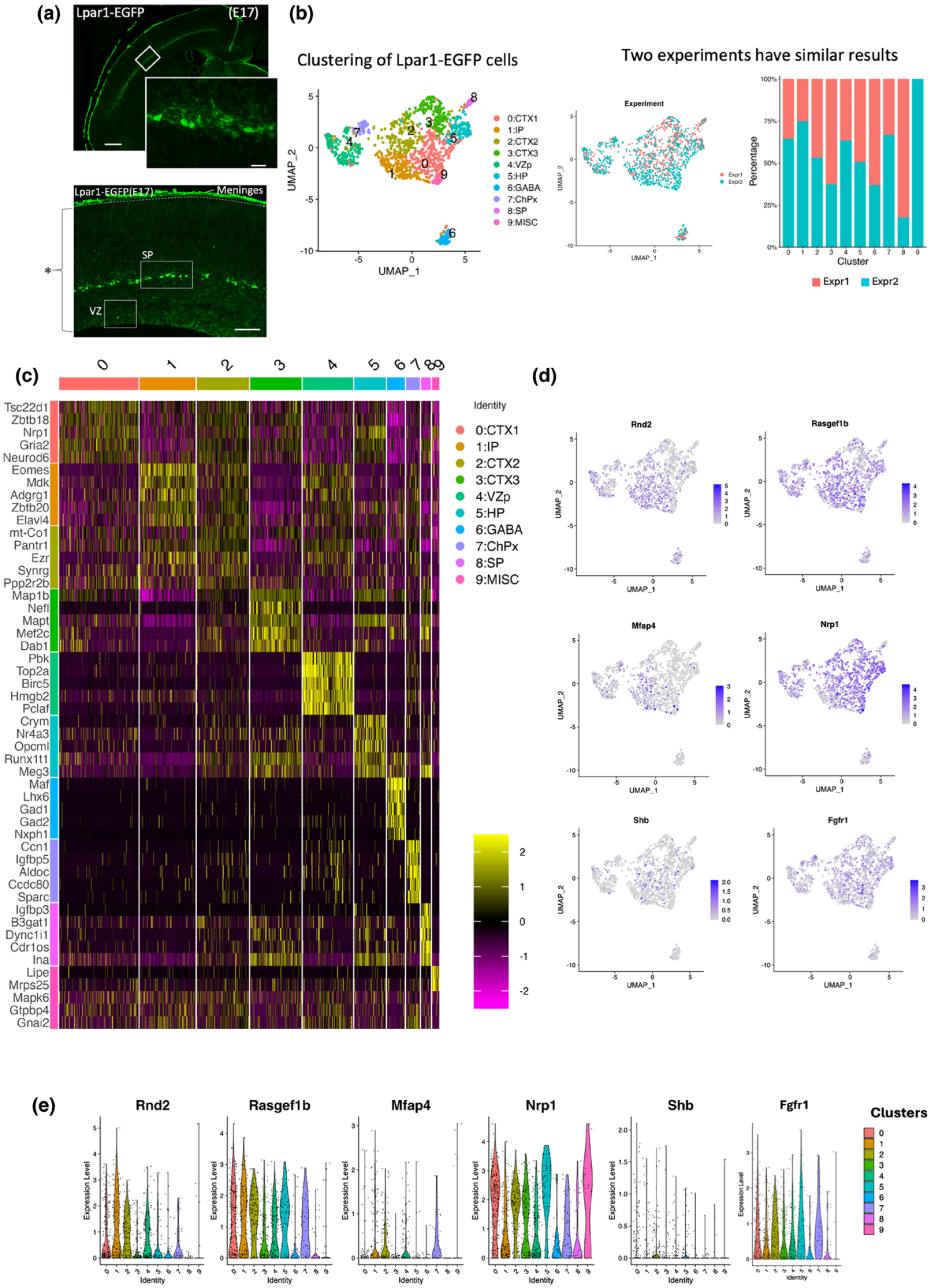
Analysis of single‐cell RNA seq of Lpar1‐EGFP‐positive cells in the 17‐day embryonic cerebral cortex. (a) (Upper panel) Coronal sections of Lpar1‐EGFP mouse cerebrum. Subplate neurons are EGFP‐positive. The pia mater expressed EGFP, but no EGFP‐positive cells were found in the cortical plate at this stage. High‐power inset shows the polygonal morphology of the EGFP‐positive subplate cells. (Lower panel) a higher‐magnification view of the cortical wall. The asterisk indicates the cortical wall region spanning from the ventricular zone to the pial surface. Boxed areas highlight representative GFP‐positive cells in the subplate (SP) and ventricular zone (VZ). Scale bars: 200 μm for the upper panel, 20 μm for the inset, and 100 μm for the lower panel. (b) UMAP plot of FACS‐sorted EGFP‐positive subplate cells revealed 10 clusters at E17. The clusters are color‐coded. On the right, the results of two independent experiments are superimposed. (c) Heatmap showing the expression of the top five most highly expressed genes in each cluster. (d) Feature plots of the six genes identified from microarray data. (e) Violin plots of these six genes. ChPx, choroid plexus; CTX, cortex; GABA, GABAergic neuron; HP, hippocampus; IP, intermediate progenitor; MISC, miscellaneous; SP, subplate neuron; VZp, ventricular zone progenitor.

At E17, Lpar1‐EGFP‐labeled neurons were predominantly located in the SP region (Figure 2a) and were absent from the cortical plate and marginal zone. The lower panel of Figure 2a shows a higher‐magnification view of the cortical wall, illustrating the distribution of EGFP‐positive cells in the VZ and SP. EGFP‐positive cells were also observed in the pia mater, corresponding to meningeal cells, but not within the cortical tissue. The EGFP‐positive SpNs exhibited a polygonal morphology (Figure 2a, inset). A few faintly labeled cells were present in the VZ (see boxed region in the lower panel of Figure 2a), but somatostatin‐immunoreactive GABAergic neurons, which appear postnatally (Marques‐Smith et al., 2016), were not yet detected.

To comprehensively characterize these SpNs, we performed single‐cell RNA sequencing on FACS‐sorted EGFP‐positive cells from Lpar1‐EGFP mice at E17 and obtained gene expression profiles of 510 cells for Expr1 and 696 cells for Expr2. We used UMAP to visualize the single‐cell RNA sequencing data, where each data point represents a cell and multiple genes are measured. The UMAP of the FACS‐sorted EGFP‐positive SP cells revealed 10 clusters at E17 (0–9) (Figure 2b). We obtained highly similar clustering results under two independent experimental conditions (Expr1‐red and Expr2‐light blue), and the substantial overlap between datasets indicated high reproducibility. It is reassuring that the two separate experiments covered similar cell type distribution (Figure 2b).

We identified clusters as heatmaps based on their gene expression; we constructed a heatmap showing the expression of the top five most highly expressed genes in each cluster (Figure 2c): Cl.0: CTX1(Cortex)‐*Zbtb18, Nrp1*; Cl.1: IP:Intermediate Progenitor—*Eomes (Tbr2), Ngn2*; Cl.2: CTX2—*Pantr1*, *Ezr*; Cl.3: CTX3—*Mef2c, Dab1*; Cl.4: VZp (VZ progenitor)—*Top2a*, *Mki67*; Cl.5: HP‐Hippocampus—*Crym, Nr4a3*; Cl.6: GABA neurons/*Lhx6*, *Gad1*; Cl.7: ChPx (Choroid plexus)—*Ccn1, Igfbp5*; Cl.8: SP (SpNs)—*Igfbp3, Nr4a2*; Cl.9: Miscellaneous (Misc)—*Lipe, Mrps2*5. The clustering results suggest potential heterogeneity among Lpar1‐EGFP‐positive spiny neurons (SpNs), while also indicating possible contamination by non‐SpNs during the sorting process. We investigated the correlation between the expression of the six genes identified through microarray analysis (*Nrp1*, *Rasgef1b, Rnd2, Mfap4, Sh*b, and *Fgfr1*) and gene expression patterns in UMAP plots derived from FACS‐sorted Lpar1‐EGFP single‐cell RNA sequencing (scRNA‐seq) data. Among these markers, *Rnd2, Mfap*4, and *Shb* exhibited elevated expression in clusters 0, 1, and 2, whereas *Rasgef1b*, *Nrp1*, and *Fgfr1* showed a broader distribution across clusters (Figure 2d). ISH data revealed robust expression of *Rasgef1b* and *Nrp1* in layers just below the SP (Figure 1b), suggesting potential contamination by neural progenitor cells from the VZ or the IZ (Figure 2a). These findings are consistent with violin plots (Figure 2e) of the six genes identified through microarray analysis (genes with FC> 2.0 in both E15 and E18 stages), which further confirm the observed expression patterns.

### 3.3 Spatial mapping of single‐cell clusters through integration with Visium data

**FIGURE 3:**
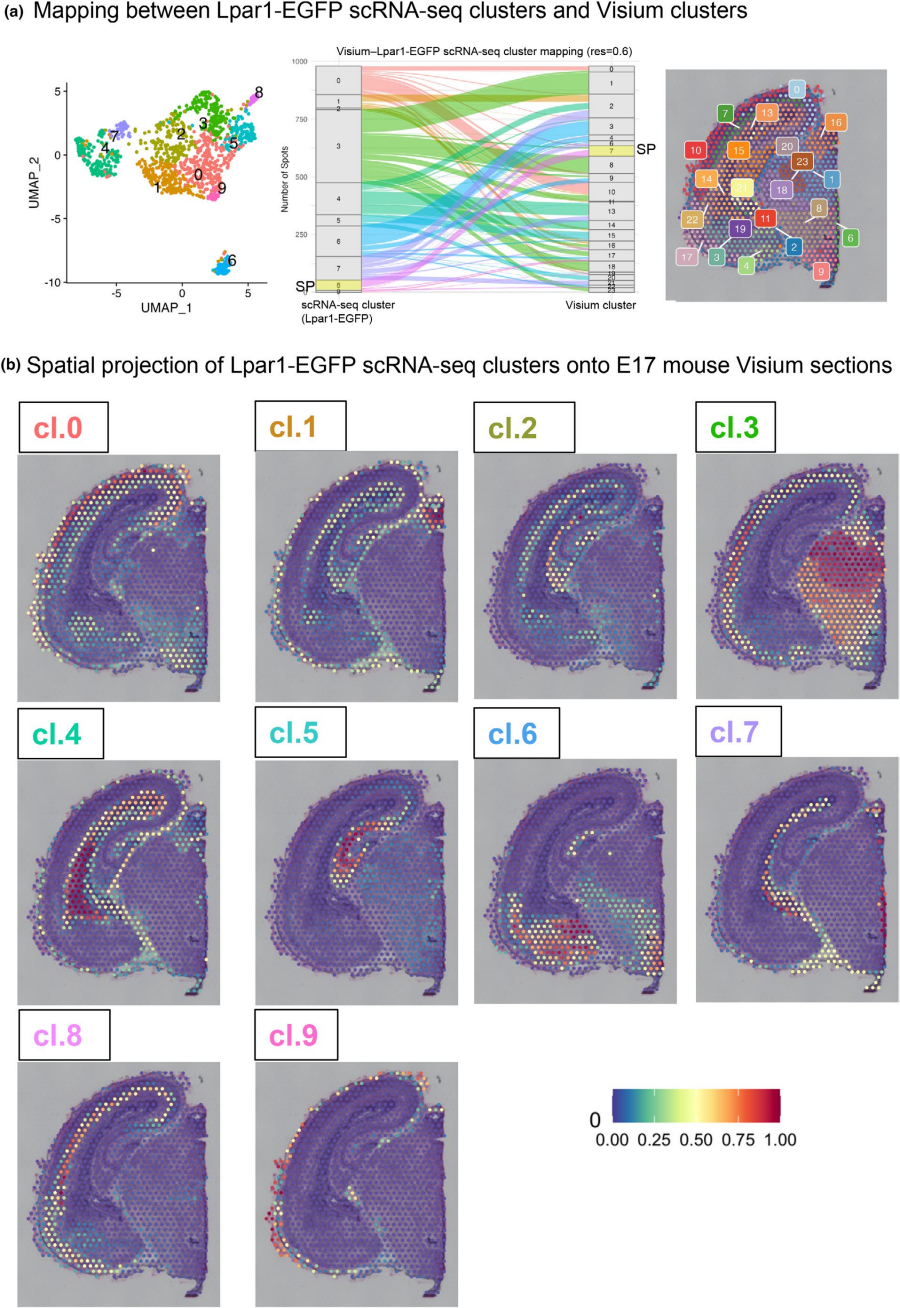
Spatial mapping of single‐cell clusters through integration with Visium data. (a) Integration of Lpar1‐EGFP+ scRNA‐seq data with E17 Visium spatial transcriptomics data using an anchor‐based workflow. Left: UMAP representation of scRNA‐seq clusters. Middle: Alluvial plot showing the correspondence between scRNA‐seq clusters and Visium clusters. Right: Spatial distribution of Visium clusters across the cortical section. The subplate (SP) region is indicated as cluster 7. (b) Spatial feature plots showing the predicted spatial distribution of individual Lpar1‐EGFP+ scRNA‐seq clusters (clusters 0–9). Color intensity represents scaled prediction scores (0–1). These maps represent projection‐based prediction scores of scRNA‐seq clusters onto Visium spots, rather than Visium‐defined clusters. Cluster 5 exhibited enrichment in the hippocampal region, cluster 6 corresponded to GABAergic neuron populations, and cluster 7 was associated with the choroid plexus. The remaining clusters were primarily associated with subplate neurons, with cluster 8 showing the most specific enrichment within the SP layer.

We have previously performed Visium spatial transcriptome analysis on the developing mouse brain (E17, P0) to analyze detailed gene expression patterns (Hara et al., 2025). To first assess the correspondence between clusters identified in the Visium dataset and those in the Lpar1‐EGFP scRNA‐seq dataset, we generated an alluvial plot. This analysis revealed that Visium SP cluster 7 showed the strongest correspondence with Lpar1‐EGFP cluster 8 (Figure 3a, middle). Using the E17 dataset, we then conducted an integrated analysis with our Lpar1‐EGFP single‐cell RNA‐seq data to investigate which regions each cluster corresponds to.

The integrated analysis revealed that the cells of Cluster 8 (Cl.8), those with the most typical expression of SpN markers, were mapped to the SP layer in the Visium data as expected (Figure 3, Cl.8). Additionally, three clusters (Cl.5: Hippocampus, Cl.6: Subcortical GABAergic, Cl.7: Choroid plexus) were associated with regions distinctly different from the SP layer. These can be interpreted as clusters of minor contaminating cells. However, the remaining clusters (Cl.0, Cl.1, Cl.2, Cl.3, Cl.4) were all mapped to regions that included the SP zone and contained SpNs. The two methodologies (single‐cell transcriptomic analysis of FACS‐sorted Lpar1‐EGFP neurons and the Visium spatial transcriptomic analysis) cross‐validated each other.

### 3.4 NeuroD1‐CreERT2 (D1B)/Ai14 labeled a partially overlapping but largely distinct population from Lpar1‐EGFP SpNs

**FIGURE 4:**
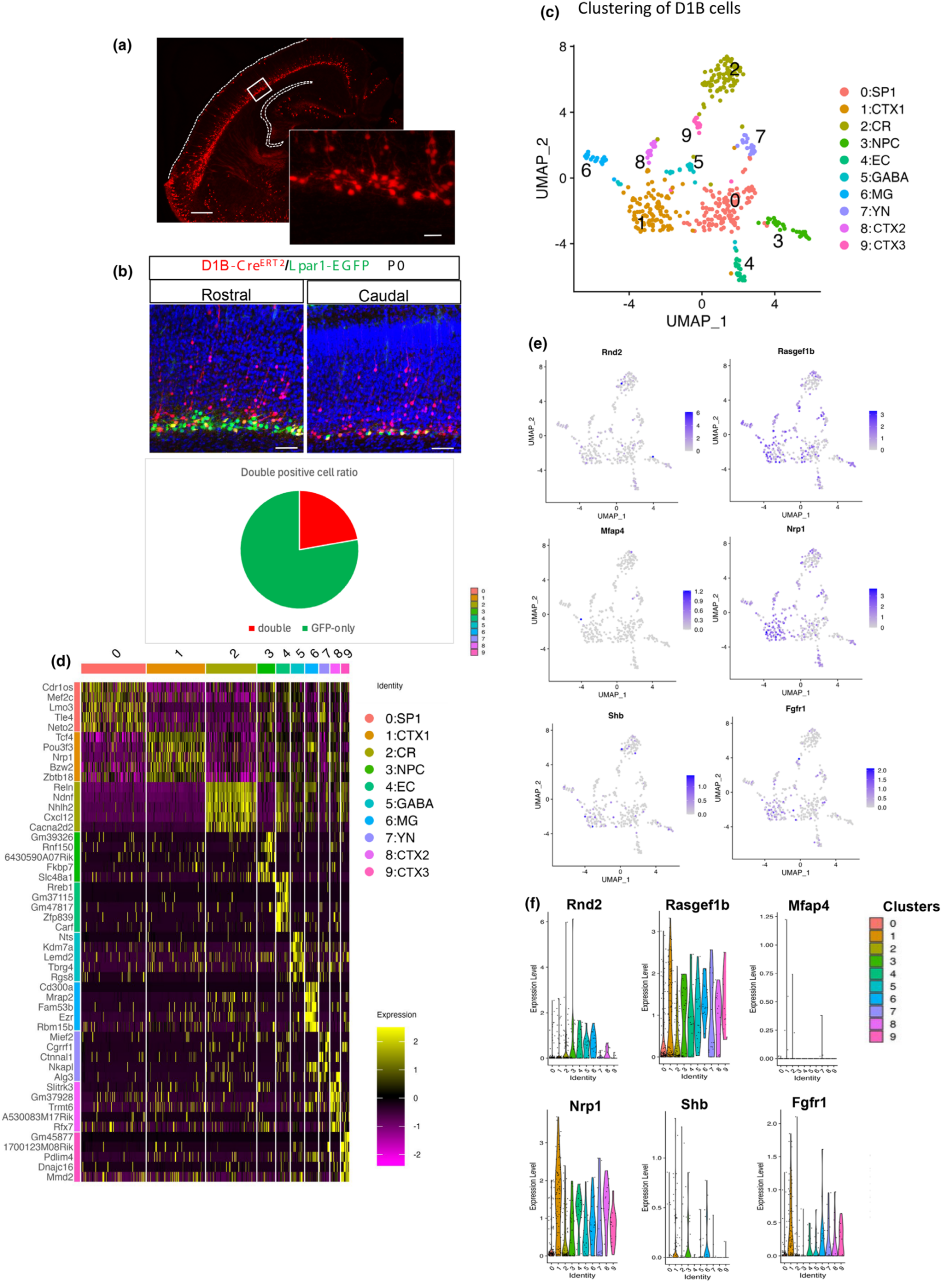
Analysis of single‐cell RNA seq of D1B‐CreERT2 positive cells in the 17‐day embryonic cerebral cortex. (a) Coronal sections of D1B mouse cerebrum. Subplate neurons are tdTomato‐positive. Some Cajal–Retzius cells expressed tdTomato, but no tdTomato positive cells were found in the cortical plate. High power insert shows the polygonal morphology of the tdTomato positive subplate cells. (b) Lpar1‐EGFP and D1B‐Cre positive tdTomato cells were double positive in 22% of cells, while the remaining 78% were single positive, indicating that they label different subpopulations. (c) UMAP plot of FACS‐sorted tdTomato positive subplate cells revealed 10 clusters at E17. The clusters are color coded. On the right, the results of two independent experiments are superimposed. (d) Heatmap showing the expression of the top five most highly expressed genes in each cluster. (e) Feature plots of the six genes identified from microarray data. (f) Violin plots of these six genes. CTX, cortex; EC, endothelial cell; GABA, GABAergic neuron; ICR, Cajal–Retzius cells; MG, microglia; NPC, neural progenitor cell; SP, Subplate neuron; YN, young neuron. Scale bars: 200 μm for a, 20 μm for the inset, and 50 μm for b.

Next, we performed sc‐RNAseq analysis using Tg mouse lines that label distinct SpN cell populations. NeuroD1‐CreERT2(D1B) driver labels layer 6b neurons following injection of tamoxifen at E11.5–12.5 (Hirata et al., 2021). We crossed Lpar1‐EGFP mice with D1B;tdTomato mice and administered tamoxifen at E10.5 to label NeuroD1‐Cre‐positive cells with tdTomato. Cell counting revealed that approximately 22% of GFP+ cells were double‐positive (Figure 4b), with the remaining cells being single‐positive for GFP. To investigate the gene expression profile of SpN distinct from Lpar1‐EGFP, we isolated tdTomato‐positive cells via FACS and performed scRNA‐seq analysis. We found 10 clusters of the FACS‐sorted tdTomato+ D1B‐CreERT2 positive SP cells (437cells) at E17 (Figure 4c). We annotated the clusters based on the expression of the top 20 highly expressed genes of each cluster(Figure 4d) as: Cl.0: SP1 (SpNs)—*Mef2c, Lmo3*; Cl.1: CTX1 (Cortex)—*Nrp1*, *Zbtb18*; Cl.2: CR (Cajal–Retzius cells)—*Reln, Calb2*; Cl.3: NPC (Neural progenitor)—*Nes, Eml1*; Cl.4: EC (Endothelial cells)—*Vegfa, Ddah1*; Cl5: GABA (GABAergic neurons)—*Nts, Rgs8*; Cl.6: MG (Microglia)—*Cd300a, Bace1*; Cl.7: YN (Young Neurons)—*Sema4g, Robo1*; Cl.8: CTX2 (Cortex)—*Nr4a2, Bhlhe22*; Cl.9: CTX3 (Cortex)—*Ttl, Mmp17*. Some of these clusters were not SP‐specific (Figure 4d). This result is consistent with expectations, as Cre‐mediated recombination is not limited to the SP layer but is also observed in some cells of the upper layers and in a small number of Cajal–Retzius cells. Feature plots and Violin plots (Figure 4e,f respectively) of the six genes identified from microarray data and validated from in situ databases (*Rnd2; Rasgef1b; Mfap4; Shb; Nrp1; Fgfr1*) showed considerable variations in expression across the different clusters.

### 3.5 Integration and comparison of single‐cell RNA‐seq data of Lpar1‐EGFP and D1B SP cells at E17

**FIGURE 5:**
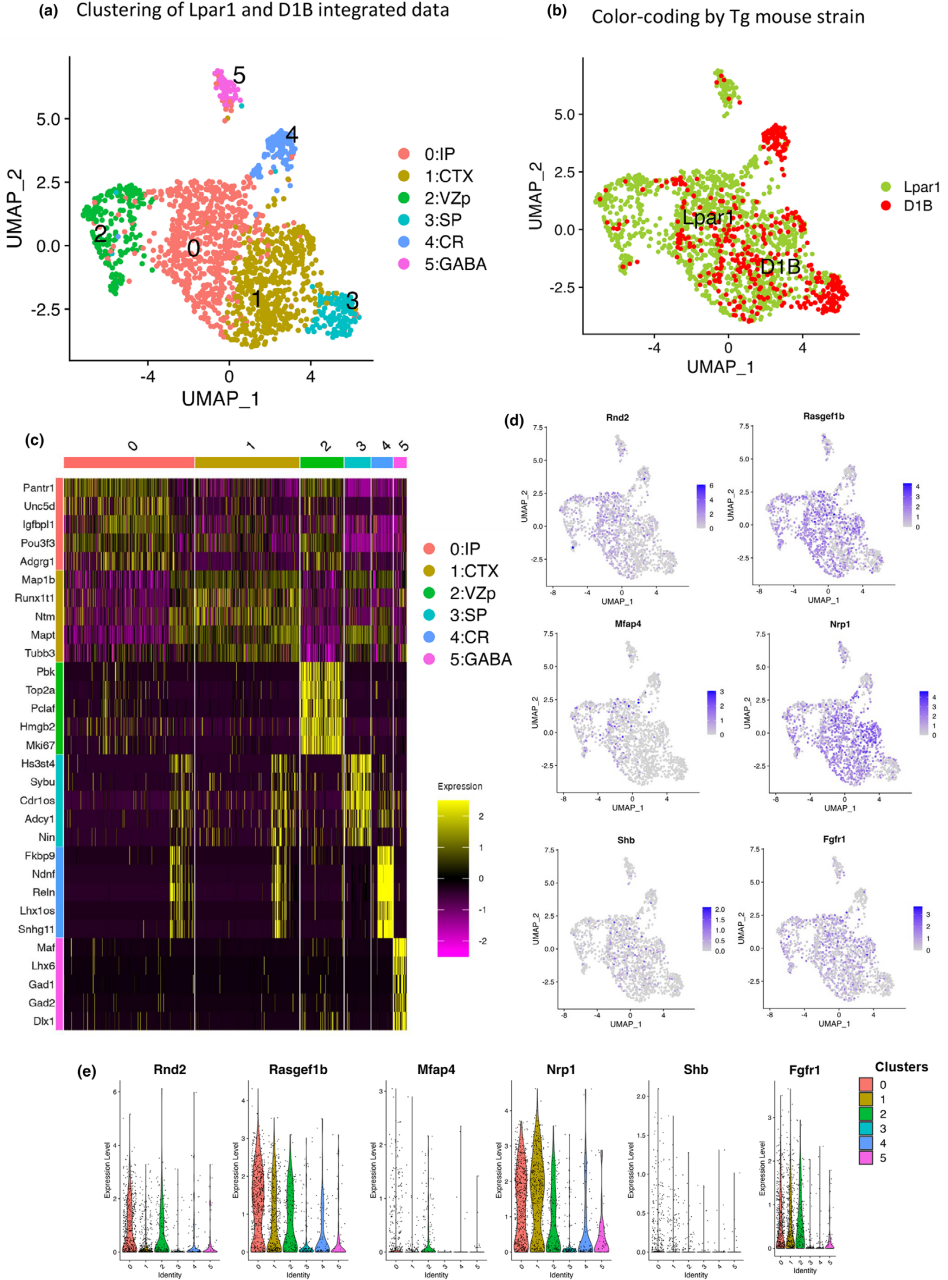
Integration of RNA‐seq data of Lpar1‐EGFP and D1B subplate cells. (a) Clustering of the integrated data of Lpar1‐EGFP and D1B cells' single‐cell RNA‐seq data. The resolution = 0.3 was used. (b)UMAP plot color‐coded by mouse strain. (c) Heatmap of each cluster. Genes are selected by top five genes of each cluster. (d) Feature plots of the six genes identified from microarray data. (e) Violin plots of these six genes. CR, Cajal–Retzius cell; CTX, cortex, VZp: ventricular zone progenitor; GABA, GABAergic neuron; IP, intermediate progenitor; SP, subplate neuron.

Subsequently, we integrated the data from two different SP models (EGFP and Td‐tomato) and explored the resulting clusters (Figure 5a). Single‐cell profiles obtained from the mouse strains (Lpar1‐EGFP and D1B ‐E17) show considerable, but not complete overlap (Figure 5b). We identified clusters as heatmaps based on their gene expression. A heatmap showing the expression of the top 20 highly expressed genes in each cluster revealed some as SP markers, but others as VZ progenitors, intermediate progenitors, Cajal–Retzius cells and GABAergic neurons (Figure 5c); Cl.0: IP (Intermediate Progenitor)—*Eomes/Tbr2, NeuroD1*; Cl.1: CTX (Cortex)—*Map1b*, *Ncam1*; Cl.2: VZp (VZ progenitor)—*Top2a, Mki67*; Cl.3: SP (SpNs)—*Hs3st4, Nr4a2*; Cl.4: CR (Cajal–Retzius cells)—*Reln*, *Cxcl12*; Cl.5: GABA neurons—*Lhx6, Gad1*. The feature and violin plots of the six genes (*Rnd2; Rasgef1b; Mfap4; Shb; Nrp1; Fgfr1*) identified from microarray data showed considerable variation in expression levels across the different clusters. We continued to explore these combined results with E17 mouse Visium data.

### 3.6 Integration of the combined single‐cell RNA‐seq data with the E17 mouse Visium data

**FIGURE 6:**
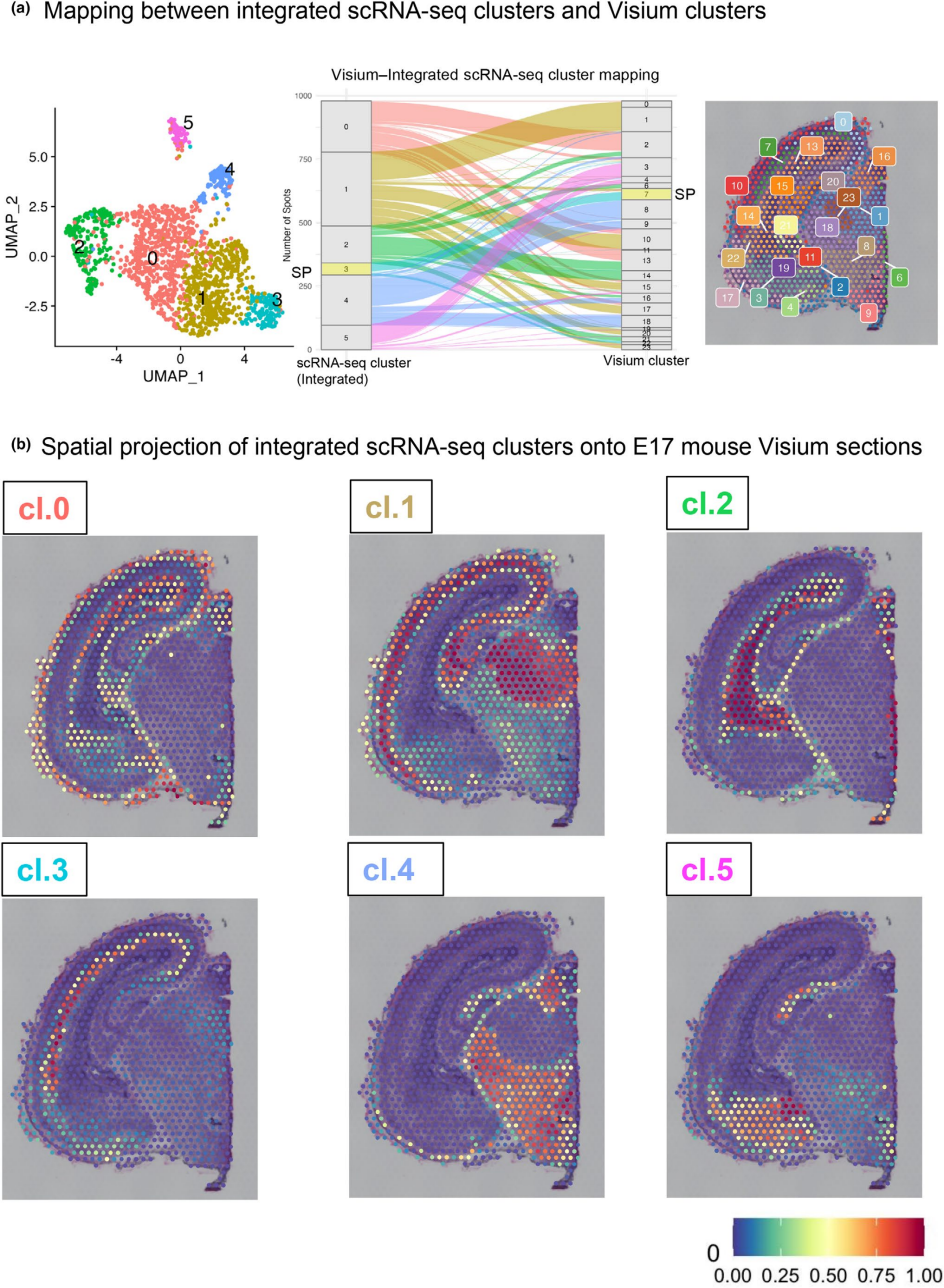
Spatial mapping of integrated single‐cell clusters through integration with Visium data. (a) Integration of the combined scRNA‐seq dataset (Lpar1‐EGFP+ and D1B‐CreERT2+ cells) with E17 Visium spatial transcriptomics data using an anchor‐based workflow. Left: UMAP representation of integrated scRNA‐seq clusters. Middle: Alluvial plot showing the correspondence between integrated scRNA‐seq clusters and Visium clusters. The subplate (SP) cluster is indicated as Cluster 7. Right: Spatial distribution of Visium clusters across the cortical section. (b) Spatial feature plots showing the predicted spatial distribution of individual integrated scRNA‐seq clusters (cl.0–cl.5). Color intensity represents scaled prediction scores (0–1). These maps represent projection‐based prediction scores of scRNA‐seq clusters onto Visium spots, rather than Visium‐defined clusters. Cluster 4 showed enrichment in the hippocampal region, and cluster 5 corresponded to GABAergic neuron populations. The remaining clusters were primarily associated with subplate neurons, with cluster 3 showing the most specific enrichment within the subplate layer.

To integrate our single‐cell RNA‐seq data with the E17 mouse Visium dataset, we applied the anchor‐based workflow (Figure 6). To first assess the correspondence between clusters identified in the Visium dataset and those in the integrated scRNA‐seq dataset, we generated a plot. This analysis revealed that Visium SP cluster 7 showed the strongest correspondence with integrated cluster 3 (Figure 6a, middle). Using the Visium E17 dataset, we then conducted an integrated analysis with our integrated single‐cell RNA‐seq data to investigate which regions each cluster corresponds to. This analysis identified six clusters. Clusters 4 and 5 showed high expression in the hippocampus and GABAergic neurons, respectively, whereas clusters 0, 1, 2, and 3 were all partially associated with SpNs. Among them, cluster 3 showed the most specific expression in the SP layer.

### 3.7 Overlap of top marker genes for SpNs from each analysis and their spatial validation

**FIGURE 7:**
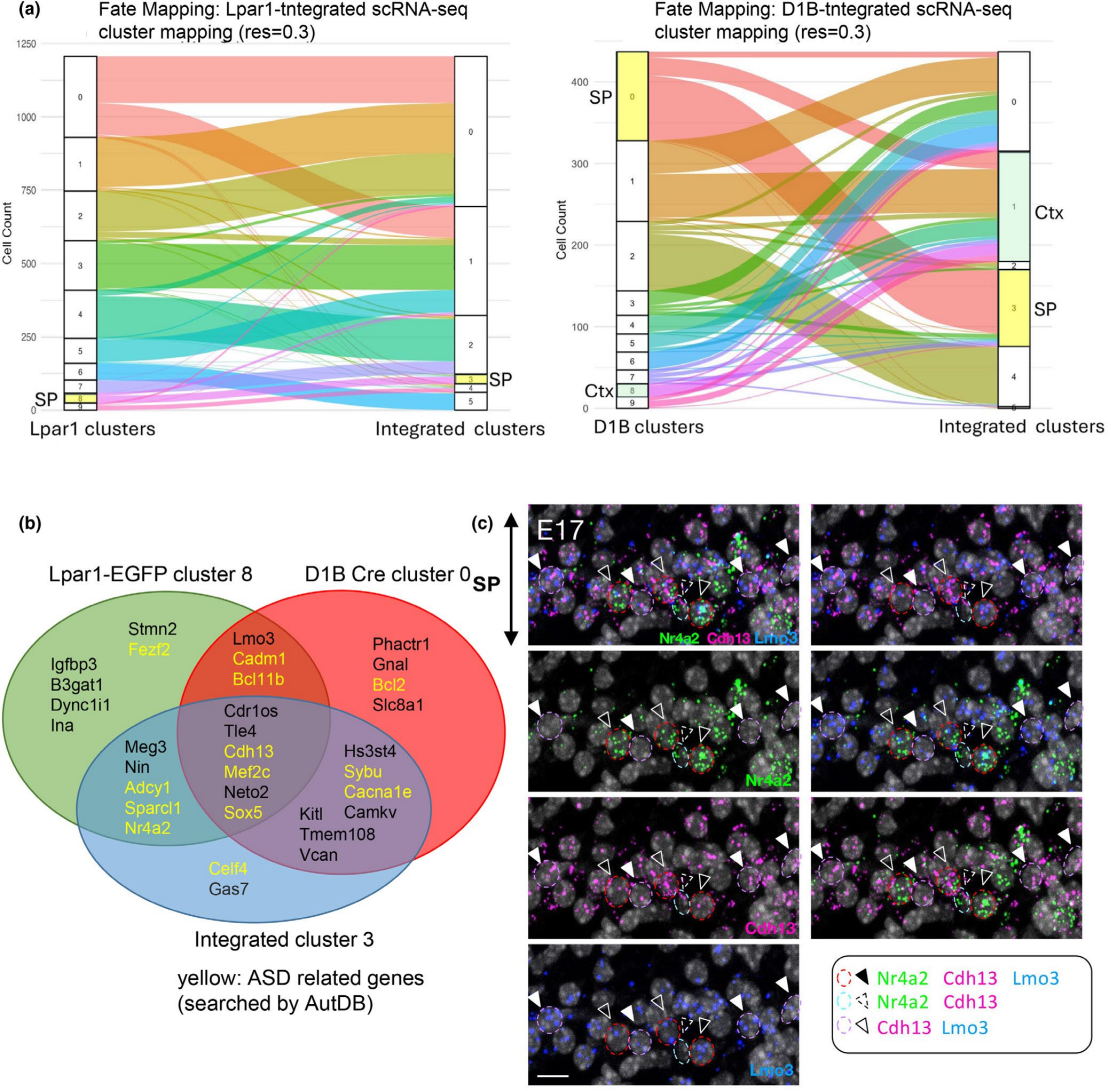
Correspondence of subplate neuron clusters across datasets and their molecular and spatial validation (a) Alluvial plots showing the correspondence between individual dataset‐specific clusters and integrated clusters. Left: Mapping of Lpar1‐EGFP+ scRNA‐seq clusters to integrated clusters. Right: Mapping of D1B‐CreERT2+ scRNA‐seq clusters to integrated clusters. These analyses clarify how subplate‐related clusters identified in each dataset (e.g., Lpar1 cluster 8 and D1B cluster 0) are represented in the integrated dataset and demonstrate that integrated cluster 3 captures the major subplate neuron populations. (b) Venn diagram illustrating the overlap of the top 20 marker genes identified from three subplate‐related clusters: Lpar1‐EGFP+ cluster 8, D1B‐CreERT2+ cluster 0, and integrated cluster 3. Genes highlighted in yellow are associated with autism spectrum disorder (ASD) based on AutDB. (c) RNAscope in situ hybridization of representative subplate marker genes (Nr4a2, Cdh13, and Lmo3) in the E17 mouse cortex. Subplate neurons are classified into molecularly distinct subtypes based on combinatorial expression patterns, including triple‐positive and double‐positive populations. Arrowheads indicate representative labeled cells. Scale bar, 10 μm.

The top 20 marker genes for the SpN clusters identified in each dataset are listed in Table 2. To clarify the correspondence between dataset‐specific clusters and the integrated clusters, we performed cluster mapping analysis (Figure 7a). This analysis shows that Lpar1‐EGFP cluster 8 and D1B‐Cre cluster 0 predominantly map to integrated cluster 3, supporting the use of these clusters for comparative analysis. Next, we created a Venn diagram showing the overlap among the top 20 marker genes identified from single‐cell RNA sequencing (scRNA‐seq) analyses of three datasets: Lpar1‐EGFP+ SpNs (Cluster 8), D1B‐Cre + tdTomato+ SpNs (Cluster 0), and Cluster 3 from the integrated dataset (Figure 7b). The 33 genes were divided into seven subtypes according to the overlap in expression. Genes in yellow are those reported to be associated with autism (Figure 7b). We selected three genes—*Cdh13, Nr4a2, and Lmo3*—from the list and examined their co‐expression at the cellular level using RNAscope. We observed triple‐positive, double‐positive, and single‐positive cells, confirming the presence of distinct cellular subtypes (Figure 7c).

**TABLE 2:** Top 20 marker genes for subplate clusters in each dataset.

par1‐cl.8	D1B‐cl.0	Integrated‐cl.3
Igfbp3	Cdr1os	Hs3st4
B3gat1	Mef2c	Sybu
Dync1i1	Lmo3	Cdr1os
Cdr1os	Tle4	Adcy1
Ina	Neto2	Nin
Meg3	Sybu	Cacna1e
Adcy1	Cadm1	Tle4
Lmo3	Sox5	Cdh13
Nin	Cdh13	Mef2c
Nr4a2	Cacna1e	Meg3
Stmn2	Hs3st4	Camkv
Sox5	Bcl11b	Neto2
Cadm1	Phactr1	Celf4
Bcl11b	Gnal	Sox5
Neto2	Camkv	Sparcl1
Sparcl1	Vcan	Kitl
Cdh13	Kitl	Gas7
Mef2c	Bcl2	Nr4a2
Tle4	Slc8a1	Tmem108
Fezf2	Tmem108	Vcan

*Note*: Lists of the top 20 genes enriched in subplate neuron clusters identified in each dataset. The lists correspond to cluster 8 of the Lpar1‐EGFP dataset, cluster 0 of the D1B dataset, and cluster 3 of the integrated analysis. Genes detected in two or more datasets are highlighted in orange.

### 3.8 Expression patterns of SpN markers identified in this study during the embryonic stage in the mouse cerebral cortex

**FIGURE 8:**
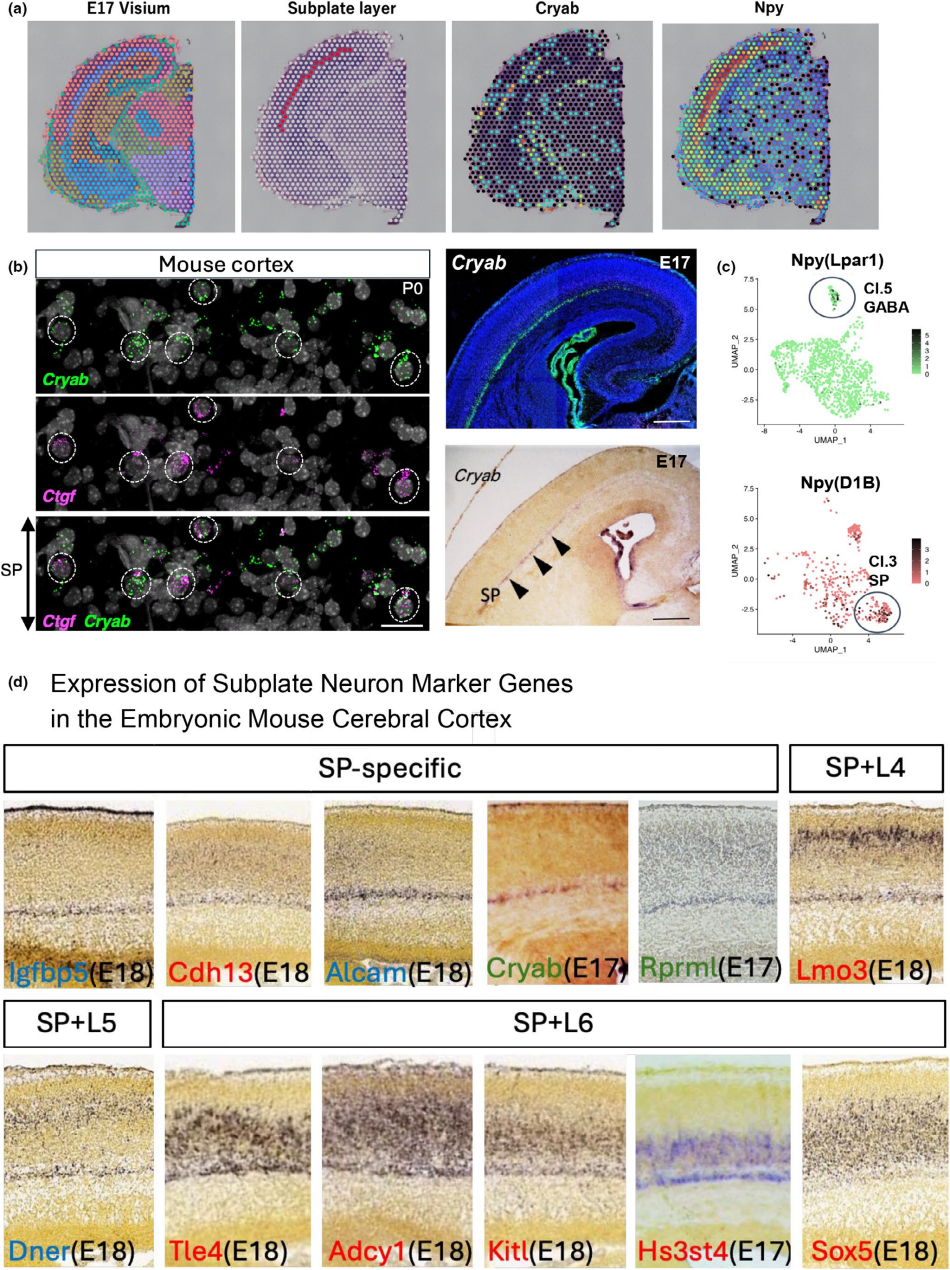
Expression patterns of subplate neuron markers identified in this study during the embryonic stage in the mouse cerebral cortex. (a) Visium spatial transcriptomics analysis identified highly expressed genes in subplate cells (Table 3). Cryab and Npy were the top two genes from this list. (b) In mice, Cryab expression was specifically detected in CTGF‐positive subplate cells using in situ hybridization and RNAscope. Scale bars: 20 μm for the left panel and 200 μm for the middle panels. (c) Feature plots showing Npy expression on D1B and Lpar1 cell clusters. UMAP visualization of the integrated analysis clustering (Figure 5b) is represented separately as a lineage‐specific plot. Green represents Lpar1‐EGFP and red represents D1B clusters. Npy‐expressing Lpar1‐EGFP+ cells are classified into the GABAergic neuron cluster (cluster 5 in the integrated dataset), while Npy + D1B cells are assigned into the subplate neuron cluster (cluster 3 in the integrated dataset). (d) Genes shown in red are included in the Venn diagram, genes in blue are listed in cluster 7 of the Lpar1‐EGFP scRNA‐seq data, and genes in green were identified through Visium analysis. The E18 images are from the Allen Brain Atlas Developing Mouse Brain. The E17 images are from in situ hybridization performed in the present study.

Finally, we identified SP‐specific marker genes during development by integrating scRNA‐seq and Visium spatial transcriptomics analyses. Table 3 is a list of genes specifically and highly expressed in the SP layer based on Visium data(Figure 8a, Table 3). Among these, we focused on *Cryab* and *Npy*. Cryab is a known marker of truncated radial glia (tRG) in the human brain (Nowakowski et al., 2016). In this study, we found that *Cryab* expression was specifically detected in *CTGF*‐positive SP cells in mice using ISH and RNAscope (Figure 8b). Neuropeptide Y (Npy) has traditionally been recognized as a marker for GABAergic neurons, particularly within the SP, where it was thought to be predominantly expressed in GABAergic SpNs (Allendoerfer & Shatz, 1994; Friauf et al., 1990). However, recent studies have revealed that in the fetal primate visual cortex (V1), Npy is also expressed in excitatory neurons (Qian et al., 2025). To further explore this point, we analyzed the integrated data to determine *Npy* expression across clusters by lineage. The results showed that in the Lpar1‐EGFP lineage, Npy is expressed in the GABAergic cluster (integrated cluster 5), whereas in the D1B lineage, it is expressed in the SpN cluster (integrated cluster 3), but not in the GABAergic cluster (Figure 8c). These findings indicate that Npy is expressed in distinct cell populations during mouse embryonic development, encompassing both GABAergic neurons and a subset of excitatory neurons.

**TABLE 3:** Top 10 genes enriched in the subplate (E17 Visium).

Gene	Log_2_ fold change	*p*
Npy	2.787426812	8.69E‐06
Cryab	2.161900578	0.39641474
Nxph3	2.161651687	0.05363365
Wnt7b	1.881362251	0.14815797
Tshz3	1.80008424	0.39641474
Rasl11b	1.796560684	0.52534954
Hs3st2	1.762452155	0.87444192
Nfe2l3	1.733773079	0.87444192
Igfbp3	1.717782203	0.52534954
Hs3st4	1.674394532	0.52534954

*Note*: Visium data from E17 mouse embryos were analyzed to identify the top 10 genes enriched in the subplate (SP), as highlighted in red in the middle panel of Figure 8a.

We further validated the expression of these genes using ISH images from the Allen Brain Atlas, showing the expression patterns of identified SpN marker genes in E17.5 or E18.5 mouse brain sections (Figure 8d). ISH data for *Cryab, Hs3st4*, and *Rprml* were generated in the present study.

Genes shown in red are included in the Venn diagram, and genes in green were identified through Visium analysis. As shown in Table 4, the genes in blue are included in the top 20 marker genes of cluster 7, which was identified as a subpopulation of the broader CTX3 cluster following higher‐resolution clustering analysis of the Lpar1 dataset (res = 2.0; see Figure S1) and showed enrichment of SpN markers. We found that some genes were SP‐specific (*lgfbp5, Cdh13, Alcam, Cryab, Rprml*); some were expressed by both SP and layer 6 (*Tle4, Adcy1, Kitl, Hs3st4, Sox5*), while *Lmo3* was expressed in layer 4, and *Dner* was expressed in layer 5 in addition to SP. These ISH results validated our combined approach across different SP populations.

**TABLE 4:** Lpar1‐EGFP+ scRNA‐seq data resolution = 2 cL.7(SPcluster)gene list.

p_val	avg_logFC	Pct.1	Pct.2	p_val_adj	Gene
2.54E‐42	1.089	0.659	0.125	5.52E‐38	Nefl
5.22E‐25	0.809	1	0.987	1.13E‐20	Map1b
8.72E‐24	1.098	0.683	0.256	1.89E‐19	Dner
2.36E‐22	0.897	0.512	0.138	5.12E‐18	Kitl
4.89E‐21	0.840	0.976	0.706	1.06E‐16	Ina
6.05E‐21	0.816	0.988	0.882	1.31E‐16	Mapt
1.40E‐20	0.862	0.988	0.784	3.05E‐16	Gap43
9.15E‐20	0.972	0.939	0.651	1.99E‐15	Meg3
9.90E‐20	0.984	0.854	0.497	2.15E‐15	Mef2c
5.78E‐19	0.885	0.902	0.6	1.26E‐14	Grin2b
6.95E‐18	1.464	0.707	0.359	1.51E‐13	Nefm
1.17E‐17	0.833	0.963	0.769	2.53E‐13	Sobp
1.28E‐16	0.691	1	0.862	2.78E‐12	Stmn2
1.12E‐14	0.680	0.878	0.601	2.44E‐10	Camk2b
1.66E‐13	0.781	0.939	0.793	3.60E‐09	Ntm
2.81E‐13	0.676	0.476	0.175	6.10E‐09	Sphkap
4.88E‐12	0.926	0.573	0.278	1.06E‐07	Alcam
5.12E‐12	0.811	0.939	0.791	1.11E‐07	Syt4
1.36E‐07	0.741	0.341	0.147	0.00295	Tshz2
2.53E‐06	0.797	0.537	0.352	0.0551	Cdh13

*Note*: The table lists the top 20 genes expressed in Nr4a2‐positive Cluster 7, identified through higher‐resolution clustering analysis (resolution = 2.0) of the Lpar1 dataset. Cluster 7 corresponds to a subpopulation of the broader CTX3 cluster identified at resolution 0.6 and shows enrichment of subplate neuron markers (see Figure S1). Genes highlighted in red were confirmed to be expressed in the subplate layer.

## 4 DISCUSSION

The molecular and genetic taxonomy of layer 6b neurons in the adult brain is increasingly well defined (Feldmeyer, 2023; Tasic et al., 2018), but our understanding of their developmental origins remains incomplete (Henning et al., 2023). This gap reflects the complexity and dynamic nature of the SP zone, which contains a changing mixture of resident neurons and transiently migrating cells. In addition, developmental maturation leads to substantial temporal changes in gene expression, further complicating the identification of stable markers. Together with cortical expansion and possible selective cell death, these processes progressively reshape the cellular composition of the SP during development. As a result, few markers are consistently expressed across developmental stages—Nr4a2/Nurr1 being a notable exception—making it difficult to monitor and manipulate SP populations using a single genetic line.

Previous transcriptomic analyses largely examined dissected tissues of whole SP layers across multiple stages (E15, E18, P8, P56), yielding only limited insight into continuous marker expression (Belgard et al., 2011; Hoerder‐Suabedissen et al., 2009; Hoerder‐Suabedissen & Molnar, 2013; Oeschger et al., 2012). *Drd1a‐Cre* or *Ctgf‐Cre* are useful for studying SP derivatives, but only after birth (Hoerder‐Suabedissen et al., 2018; Zolnik et al., 2024). Birthdating and lineage studies combined with marker analyses will be essential for tracing the developmental trajectories and survival of distinct SP populations with a single genetic line (Hoerder‐Suabedissen & Molnar, 2013; Vasistha et al., 2015).

Our study provides a novel contribution by performing transcriptomic profiling of embryonic SpNs at three timepoints (E15, E17, and E18), using three complementary approaches: microarray analysis, scRNA‐seq, and Visium spatial transcriptomics. Importantly, cross‐validation across methods increased confidence in our findings. Notably, some markers were detected preferentially in one platform but appeared weaker or more broadly distributed in their expression in other datasets. We interpret these differences as reflecting both biological heterogeneity within the embryonic SP and inherent technical characteristics of each method. Biologically, the SP is not a uniform population but comprises transcriptionally distinct subclasses. For example, genes such as *Cryab*, *Cdh13*, and *Hs3st4* were enriched in specific SP clusters rather than uniformly expressed across all SpNs, suggesting molecularly distinct subtypes. Similarly, lineage‐dependent differences in *Npy* expression across reporter lines indicate dynamic and context‐dependent transcriptional regulation during development.

From a technical perspective, each platform captures different aspects of gene expression. Microarray analysis reflects averaged expression across pooled FACS‐sorted SP‐enriched populations and may dilute signals from rare subpopulations or minor contamination from adjacent progenitor populations. In contrast, single‐cell RNA sequencing is subject to dropout effects and limited detection of low‐abundance transcripts. Spatial transcriptomics (Visium) provides anatomical context but has limited spatial resolution (~55 μm in diameter per spot), resulting in spatial averaging of multiple neighboring cell types.

Accordingly, partial discordance across platforms likely reflects complementary strengths and limitations rather than methodological inconsistency. While microarray and scRNA‐seq showed partial convergence, spatial transcriptomics confirmed the anatomical localization of SP clusters and also revealed overlapping expression in adjacent regions such as the hippocampus and marginal zone, highlighting both regional specificity and broader developmental distribution of these markers.

It should also be noted that genes identified through microarray analysis represent candidates enriched in the FACS‐isolated Lpar1‐EGFP‐positive population relative to the cortical plate, rather than markers strictly restricted to the SP. Consistent with this interpretation, public ISH datasets indicate that some candidates show broader or developmentally dynamic expression patterns. For example, *Mfap4* is detected in the preplate/SP at early embryonic stages but appears more prominently in the VZ at later stages in the Allen Brain Atlas.

These observations suggest that some genes identified here mark SP‐enriched embryonic populations or temporally dynamic transcriptional states rather than spatially exclusive SP identities. Partial differences across datasets, therefore, likely reflect the complementary detection modalities and cellular compositions of microarray and public ISH resources.

By isolating SpNs from two reporter mouse lines—*Lpar1‐EGFP* and *NeuroD1‐CreERT2 (D1B)*—we were able to define overlapping and distinct transcriptional profiles. Integrated analysis revealed Cluster 8 (*Lpar1‐EGFP*), Cluster 0 (D1B‐Cre), and Cluster 3 (combined dataset) as the clusters most enriched for SP identity. Validation with ISH and RNAscope confirmed the selectivity of key markers, including *Cryab*, *Cdh13*, *Nr4a2*, and *Lmo3*. It should also be noted that additional factors may contribute to the incomplete overlap between the two labeled populations. The Lpar1‐EGFP and NeuroD1‐CreERT2 mouse lines are maintained on different genetic backgrounds, and the Lpar1‐EGFP transgene is located on the Y chromosome, resulting in EGFP expression only in male embryos. These factors may partially influence the representation of labeled cells and should therefore be considered when interpreting the comparison between the two reporter lines.

Our analysis identified genes differentially enriched across embryonic SP clusters that may represent candidate markers of transient or persistent SpN populations. Because the cells analyzed here were genetically labeled and isolated by FACS using Lpar1‐EGFP or NeuroD1‐CreERT2; Ai14 reporter lines, the dataset is expected to be enriched for SpNs and their lineages, making it unlikely that the observed transcriptional signatures arise from neurons merely passing through the SP during migration. Instead, these expression patterns more likely reflect either molecular differences between SpN populations with distinct developmental fates or temporal gene expression changes within the same lineage during maturation. Distinguishing between these possibilities will require future lineage‐tracing and temporal analyses across developmental stages. Interestingly, several of the marker genes shared across SP clusters are also listed in the Autism Database (AutDB). Although the functional significance of this overlap remains to be determined, it is consistent with previous studies suggesting that the SP represents a key developmental stage in cortical circuit formation that may be vulnerable to neurodevelopmental perturbations (Hoerder‐Suabedissen et al., 2013).

CRYAB (αB‐crystallin) is a small heat‐shock protein that functions as a molecular chaperone to prevent protein misfolding and protect cells from stress. Its neuroprotective and stress‐responsive roles are consistent with the importance of the HSF‐regulated heat‐shock pathway in brain development and neural cell homeostasis. For example, *HSF2 is required for cortical developmen*t (Chang et al., 2006), *and interactions between HSF1 and HSF2 modulate stress‐responsive gene expression* (El Fatimy et al., 2014; Roos‐Mattjus & Sistonen, 2022). *HSF2 deficiency causes neuronal migration defects, whereas prenatal stress alters HSF1–HSF2 interactions, suggesting that these factors coordinate stress responses and corticogenesis*. The identification of *Cryab* expression in mouse SpNs is particularly intriguing. In the human fetal cortex, CRYAB marks truncated radial glia (tRG), which are not present in rodents (Nowakowski et al., 2016). In human brain development, CRYAB is expressed in the mid‐fetal cerebral cortex in shortened radial glial cells (tRG cells), early oligodendrocyte precursor cells, and some stellate glial cells. Unlike mice, tRG cells are located in the VZ and are characterized by processes that contact only the ventricular surface, which is unique to humans and other gyrencephalic species, including ferret (Bilgic et al., 2023) and macaque (Nowakowski et al., 2016). However, the presence of tRG cells has not been reported in mice. In this study, we found that *cryab* is specifically expressed in the SP layer of the mouse fetal brain. The reason for this is unclear, but there may be some commonalities between tRG in primates and SP cells in the mouse developmental stage. Further investigation of this may shed light on the mysteries of cerebral cortex formation.

Another notable marker, Neuropeptide Y (*Npy*), was highly expressed in a subset of inhibitory SP interneurons. In our single‐cell dataset, *Npy* expression was also detected in a small subset of excitatory neurons. These cells did not show transcriptional signatures consistent with doublets or progenitor contamination based on standard quality‐control metrics and marker gene expression. RNAscope analysis further confirmed the presence of *Npy*‐positive cells within the SP region at E17, consistent with the pattern observed in the single‐cell dataset. Because the dissected tissue samples were obtained from the dorsal pallium and did not include the primary visual cortex region, contamination from V1‐derived neurons is unlikely to account for this observation.

Prior studies in cat visual cortex and rodent SP have implicated NPY in neuronal migration, differentiation, and early circuit formation (Chun & Shatz, 1989a, 1989b; Hoerder‐Suabedissen & Molnar, 2013, 2015; Wahle et al., 1987). Through Y1 receptors and ERK signaling, NPY may also promote neuroprotection and progenitor cell proliferation (Decressac & Barker, 2012). However, NPY expression is not limited to inhibitory neurons and occurs in other cell types, such as progenitors and non‐GABAergic neurons, during development (Chun & Shatz, 1989b; Hoerder‐Suabedissen & Molnar, 2015; Wahle et al., 1987). Its broader developmental expression across multiple cell types suggests that NPY may contribute not only to local inhibitory signaling but also to circuit stabilization and neuroprotection during SP maturation (Li et al., 2019).

In summary, by integrating microarray, single‐cell RNA sequencing, and spatial transcriptomics datasets, including single‐cell analyses across two reporter lines with robust prenatal expression, we identify both shared and lineage‐specific gene expression signatures that define early distinct SP subpopulations and provide a framework for future studies of their roles in early cortical circuit formation and neurodevelopmental disorders. Combining these newly identified markers with birthdating and fate mapping studies shall enable us to distinguish transient and more permanent populations of SpNs.

## Supporting information


**Figure S1.** Comparison of clustering results of Lpar1‐EGFP cells at different clustering resolutions.

Data S1.

## Data Availability

The data that support the findings of this study are available from the corresponding author upon reasonable request.
