# Supplementary material for: Transcriptomic profiling of subpopulations of mouse embryonic subplate neurons
Source: J Anat. 2026 Jul 16:10.1111/joa.70197. Online ahead of print. doi: 10.1111/joa.70197 (PMC13398646; doi:10.1111/joa.70197)
Supplement: Supplementary file 1 — Figure S1. Comparison of clustering results of Lpar1‐EGFP cells at different clustering resolutions. [file JOA-9999-0-s001.pptx]

## Slide 1
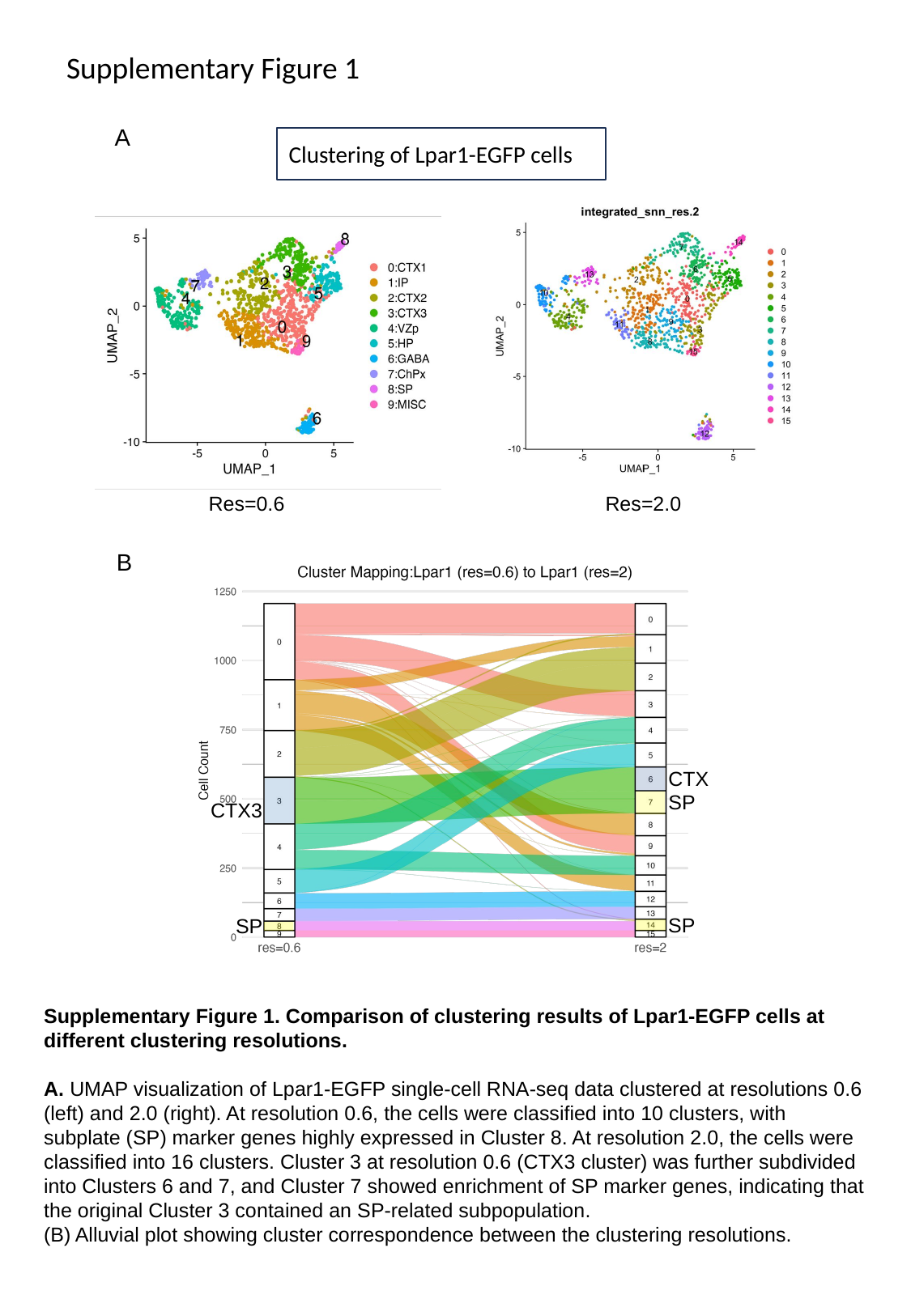

Supplementary Figure 1
A
Clustering of Lpar1-EGFP cells
Res=0.6
Res=2.0
B
CTX
SP
CTX3
SP
SP
Supplementary Figure 1. Comparison of clustering results of Lpar1-EGFP cells at different clustering resolutions.
A. UMAP visualization of Lpar1-EGFP single-cell RNA-seq data clustered at resolutions 0.6 (left) and 2.0 (right). At resolution 0.6, the cells were classified into 10 clusters, with subplate (SP) marker genes highly expressed in Cluster 8. At resolution 2.0, the cells were classified into 16 clusters. Cluster 3 at resolution 0.6 (CTX3 cluster) was further subdivided into Clusters 6 and 7, and Cluster 7 showed enrichment of SP marker genes, indicating that the original Cluster 3 contained an SP-related subpopulation.
(B) Alluvial plot showing cluster correspondence between the clustering resolutions.
